# Mismatch uracil DNA glycosylase (Mug) is maintained in the *Corynebacterium pseudotuberculosis* genome and exhibits affinity for uracil but not other types of damage

**DOI:** 10.1590/1678-4685-GMB-2023-0353

**Published:** 2025-04-14

**Authors:** Bruno Carvalho Resende, Cássio Siqueira Souza Cassiano, Diego Lisboa Rios, Thalia Queiroz Ladeira, Vasco Ariston Carvalho Azevedo, Luciana Lara dos Santos, Lucía Valenzuela-Pérez, Gonzalo Cabrera, Carlos Renato Machado, Débora de Oliveira Lopes

**Affiliations:** 1 Universidade Federal de Minas Gerais (UFMG), Instituto de Ciências Biológicas (ICB), Belo Horizonte, MG, Brazil.; 2 Universidade Federal de São João Del-Rei (UFSJ), Divinópolis, MG, Brazil.; 3 Universidad de Chile, Faculdad de Medicina, Instituto de Ciencias Biomédicas, Santiago, Chile.

**Keywords:** Uracil, G/U mismatch-specific DNA glycosylase, Corynebaterium pseudotuberculosis, DNA repair, base excision repair

## Abstract

The genome of *Corynebacterium pseudotuberculosis*, etiologic agent of Caseous Lymphadenitis (CLA), was sequenced to comprehend its genetics, pathogenicity, and virulence mechanisms due to its economic importance. A focus was placed on the G/U mismatch-specific DNA glycosylase (Mug), an enzyme vital for base excision repair in DNA that can play an important role in uracil repair, since the high G+C content of *C. pseudotuberculosis* makes it prone to deamination events, accentuating the potential significance of Mug. Through *in silico* and *in vitro* analyses, the *Corynebacterium pseudotuberculosis* Mug protein (*Cp*Mug) was characterized to confirm its DNA glycosylase activity and lesion affinity. The *mug* gene was identified in both pathogenic and non-pathogenic Corynebacterium species, lacking a discernible ancestry pattern. Bioinformatics analyses revealed the preservation of essential uracil DNA glycosylase catalytic residues in *Cp*Mug. The 3D structure of *Cp*Mug was constructed, and molecular docking analysis demonstrated its interaction with DNA containing uracil and other lesions. Comparative analyses revealed a higher affinity of *Cp*Mug’s catalytic residues for uracil over other DNA lesions and enzymatic assays with purified *Cp*Mug affirmed its uracil DNA glycosylase activity, while it exhibited no activity on 8-oxoguanine, tetrahydrofuran, or thymine glycol, consistent with computational simulations.

## Introduction

Every living cell must deal with the constant threat of damage prevenient from environmental factors or derived from your metabolism. To ensure their survival, they depend on an intricate DNA repair system to safeguard the cell’s genetic material against harm and maintain its optimal function. UV radiation, reactive oxygen (ROS), and nitrogen (RNS) species are well-known examples of chemical and physical agents that can damage DNA molecules (Li and Sancar, 2020). The actions of these agents may result in modifications to the DNA sequence and structure, ultimately causing mutations and impairment of protein function. To prevent genetic mutations, the DNA repair system functions as a highly effective and cohesive mechanism composed of various enzymes that detect, locate, and repair DNA damage, thereby preserving the integrity of the genome. Apart from the prominent role of DNA repair in genetic maintenance, recent studies have shown non-canonical functions for this system in various cellular processes, such as dormancy, autophagy events, and pathogenesis (Gorna et al., 2010; Gomes et al., 2017; Resende et al., 2020).

Base excision repair (BER) is a DNA repair pathway conserved from bacteria to humans that recognises and corrects damaged bases that suffer oxidation, alkylation, deamination, and even single-strand breaks (SSBs), restoring the original base sequence (Krokan and Bjørås , 2013). Since ROS and RNS are the natural weapons of the innate immune system, the BER pathway has evolved in pathogenic bacterial species to protect their genomic content from these reactive species during infection, contributing to their pathogenesis (Van and Tang, 2015). The BER pathway is initiated by a specific DNA glycosylase (mono or bifunctional) that removes the damaged base, and an AP endonuclease will cleave the AP site followed by a DNA polymerase to fill the gap and a DNA ligase to seal the repaired strand (Arantes et al., 2016; Mullins et al., 2019).

In base excision repair (BER), each DNA glycosylase can identify and target particular types of base lesions with varying levels of specificity (Krokan and Bjørås, 2013). Among the spectrum of lesions recognised by BER is uracil. Every day, the DNA from all living organisms is susceptible to spontaneous cytosine deamination events, resulting in the formation of uracil. The single-strand DNA is more vulnerable to deamination than the double-strand, and the appearance of this lesion is more significant during the transcription and replication process (Owiti et al., 2019). Besides that, the misincorporation of dUTP instead of dTTP during the replication process can also result in adding uracil bases to this molecule (Lindahl and Nyberg, 1974; Chon et al., 2019).

The Uracil-DNA glycosylases prevent mutagenesis by eliminating uracil from DNA molecules by cleaving the N-glycosidic bond and initiating the base-excision repair. This enzyme is well-conserved and ubiquitous in nearly all life forms, highlighting its importance. Six uracil-DNA glycosylases (UDG) family members have been identified in human cells (Wu et al., 2022). The Thymine DNA-glycosylase (TDG) belongs to sub-family II, the human TDG, responsible for removing uracil and thymine from G:U and G:T mismatches in double-stranded DNA (dsDNA). It was first described in HeLa cells, further characterised in many other organisms, including *Escherichia coli* and *Deinococcus radiodurans* (Neddermann and Jiricny, 1994; Barrett et al., 1998; Moe et al., 2006; Schormann et al., 2014).

Apart from uracil, this glycosylase can also recognise and process other kinds of substrates, such as thymine mismatched with guanine, 1,N2-ethenoguanine, hypoxanthine, 5-hydroxycitosine, and 8-oxoadenine, showing different activity rates for each one (Talhaoui et al., 2013; Admiraal et al., 2019). 

The Mismatch-specific uracil-DNA glycosylase (Mug) is the prokaryotic counterpart of TDG. The *Escherichia coli’s* Mug (*Ec*Mug) is a thoroughly researched enzyme that consists of five conserved β-sheets and five α-helixes, and the amino acid residues crucial for the glycosylase activity have already been identified (Barrett et al., 1998). The asparagine residue in the GINPGL motif interacts and deprotonates a water molecule, which removes the deoxyuridine through the nucleophilic attack at the N-glycosidic bond. The F30 residue provides specificity through a π-π stacking interaction with uracil, and the PNPSGL residues are responsible for stabilising the protein-DNA complex, interacting with the complementary nucleotide, guanine (Barrett *et al*., 1998). 

*C. pseudotuberculosis* is a bacterium that belongs to the CMNR group (*Corynebacterium*, *Mycobacterium*, *Nocardia,* and *Rhodococcus*) and has a high G+C genome content (47-74%) (Pinto et al., 2014; Whitaker et al., 2017). This Gram-positive pathogenic species causes caseous lymphadenitis (CLA), which affects small ruminants worldwide, mainly sheep and goats (Paton et al., 1994). In horses and buffalos, *C. pseudotuberculosis* infection causes ulcerative lymphangitis. Both conditions lead to considerable economic losses due to reduced milk and meat production (Paton *et al*., 1994). During colonisation, these bacteria must survive the host immune system attack, which generates reactive oxygen and nitrogen species by phagocytic cells, aiming to disrupt pathogen molecules. The low pH and high levels of ROS/RNS can particularly affect the DNA molecule. In this sense, the base excision repair (BER) pathway plays a vital role in repairing oxidative DNA damage and avoiding bacterial death (Stefańska et al., 2010; Arantes et al., 2016; Chatterjee and Walker, 2017).

Given the significance of Uracil DNA Glycosylases as a potential target for inhibiting bacterial infections (Mechetin et al., 2020) and building upon our prior research findings, where we verified the presence of two distinct uracil DNA glycosylases in *C. pseudotuberculosis,* the present work focused on characterising *Cp*Mug and assessing its role in DNA repair. To accomplish this, we utilised bioinformatics tools to confirm the preservation of this specific gene in the Corynebacterium genus, analysed the existence of catalytic residues in *Cp*Mug, and conducted both *in vitro* and *in silico* analysis to determine its affinity for uracil and the other three lesion types.

## Material and Methods

### Phylogenetic analyses

All the DNA and protein sequences used in this study were obtained from the National Center of Biotechnology (NCBI) and CoryneRegNet repositories (Table S1) (Pauling et al., 2012). The evolutionary history of the *Corynebacterium* Mug protein was inferred using the neighbor-joining method by analysing the conserved residues sequence present in 27 *Corynebacterium* spp. (Saitou and Nei, 1987). To conduct phylogenetic analyses, we obtained the Mug amino acid sequences from the CMNR bacteria group, which includes *Corynebacterium pseudotuberculosis*, *Mycobacterium colombiense*, *Nocardia brasiliensis*, and *Rhodococcus jostii*, as well *Escherichia coli*. These sequences were included in the analyses using MEGA-X (Kumar et al., 2018). An optimal tree was drawn to scale, with branch lengths in the same units as those of the evolutionary distances used to infer the phylogenetic tree (Tamura et al., 2004). The evolutionary distances were computed using the Poisson correction method, and the units are the number of amino acid substitutions per site (Zuckerkandl and Pauling, 1965). All positions containing gaps and missing data were eliminated from the dataset. For comparison purposes, the 16S rRNA sequences were obtained to check the evolutionary distances of the *Corynebacterium* spp.

**Characterisation of *Cp*Mug and search for conserved motifs**

To investigate the preservation of uracil DNA glycosylase motifs and the associated catalytic activity residues of *Cp*Mug, we have selected four Mug proteins from Corynebacterium that exhibit over 70% similarity with *Cp*Mug. Both pathogenic and non-pathogenic species were included in the selection, which was made through a local alignment using BLAST-P software. The select sequences were used to perform multiple alignments with the *Cp*Mug protein, *Ec*Mug, and three species of the CMNR group. The alignment was conducted using T-Coffee software (Notredame et al., 2000). Physicochemical features prediction of the *Cp*Mug was investigated using the ProtParam software, and the prediction of domains and motifs was performed using InterProScan software (Gasteiger et al., 2005; Jones et al., 2014).

***Cp*Mug protein modelling through structural homology method**

We submitted the amino acid sequence of *Cp*Mug to BLASTp searches on the Protein Data Bank (PDB) to find appropriate structural templates. The target proteins were aligned with the template structure and manually curated with the support of MEGAX (Kumar et al., 2018). Using Modeller software, a set of 50,000 candidate structures for the *Cp*Mug protein was generated (Eswar et al., 2007). The ten best structures were selected based on the discrete optimised protein energy score and further evaluated for their stereochemical properties and energy profiles using PROCHECK and ProSA software (Laskowski et al., 1993; Wiederstein and Sippl, 2007). Additionally, the secondary structure elements of *Cp*Mug protein and the templates were graphically defined and visualised using UCSF Chimera software (Pettersen et al., 2004).

**Evaluation of *Cp*Mug-DNA lesion interaction**

The 3D structure of *E. coli* Mug (*Ec*Mug) was obtained from the crystallised deposited protein (PDB-1MWI) to be used as a positive control for docking calculations and compared with the previously generated *Cp*Mug (2.10). The catalytic residues interacting with the DNA molecule and lesions: uracil, 8-oxoguanine (8-oxo), thymidine glycol (Tg) and tetrahydrofolate (THF) were analysed through docking calculations using the Haddock 2.4 advanced guru interface (Van et al., 2016). Both active enzyme residues were defined based on their positional correspondence to the crystallised *Ec*Mug and passive residues, including all residues within a radius of 6.5 Å of the active ones. In the DNA molecule, only the lesion site was considered an active base in the protein-DNA interaction. All additional parameters that were used were set to the default values. The generated complexes were assessed for stereochemical conformations, cluster sizes, energy, and the DNA lesion’s position in the Mug proteins’ active site. Finally, the UCSF Chimera software was employed to compare the DNA-binding patterns of the *Ec*Mug-DNA and the proposed *Cp*Mug-DNA complex.

**Assessment of electrostatic potential surface of *Cp*Mug in interaction with DNA**

The interaction of the *Cp*Mug protein with the DNA molecule containing uracil was compared to *Ec*Mug (PDB-1MWI) using UCSF Chimera software based on alignment and measurement features (Pettersen *et al*., 2004). To assign the *Cp*Mug surface charges, the residue protonation and surface charges were examined using the PDB2PQR server, selecting the AMBER force field and internal naming scheme protocol (Dolinsky et al., 2004). The PROPKA software simulated protonation events, considering physiological pH (7.0) (Olsson et al., 2011). Results were submitted to APBS to make the macromolecular electrostatics calculations. The charged surfaces were visualised in PyMOL software (Schrödinger) using the APBS tools plugin (DeLano, 2020).

**Amplification and cloning of *Cpmug* gene**

The genomic DNA of the *C. pseudotuberculosis 1002* strain, kindly provided by Dr. Vasco Azevedo, was used as a template in the PCR to amplify the *Cpmug* gene using Promega® GoTaq® Hot-Start PCR kit following the manufacturer’s instructions. The full-length gene was amplified using the specific primers: *Cpmug*-forward 5’-GGATCCATGAGTACTGCACACCCTT-3’ and *Cpmug*-reverse 5’-GAATTCATACAGTGCGATTGTTGCG-3’; *Bam*HI and *Eco*RI restriction sites were present on the forward and reverse primers, respectively. The amplification was conducted by initial denaturation at 95 °C for 10 min, followed by 35 cycles with denaturation at 95 °C for 1 min, annealing at 54 °C for 1 min and extension at 72 °C for 1.5 min, with a 3 min final extension at 72 °C. The amplified full-length fragment was analysed by 1% agarose gel electrophoresis and stained using ethidium bromide. 

The amplified *Cpmug* was cloned into pCR-2.1 TOPO® (Invitrogen), and *E. coli* DH5α strain (dlacZ Delta M15 Delta(lacZYA-argF) U169 recA1 endA1 hsdR17(rK-mK+) supE44 thi-1 gyrA96 relA1) was transformed with the construct by electroporation and plated on 2xYT agar (1,5%) containing 100 µg/mL ampicillin and incubated at 37 °C, overnight. The pCR-2.1 TOPO-*Cpmug* was extracted from cells with the PurLinkTM Quick Plasmid Miniprep Kit (Invitrogen), submitted to digestion with *Bam*HI and *Eco*RI restriction enzymes, and the extracted insert subcloned in a pET-21a expression plasmid. The construct pET-21a - *Cpmug* was confirmed by sequencing, performed on an AB3500 Genetic Analyzer automated sequencer (Life Technology) following the equipment protocol.

**Expression and purification of recombinant *Cp*Mug**

*E. coli* Rosetta (DE3) pLysS (*E. coli* str. B F^-^ *ompT gal dcm lon*? *hsdS*_*B*_ (*r*_*B*_^-^ *m*_*B*_^-^) λ(DE3 [*lacI lacUV5*-*T7p07 ind1 sam7 nin5*]) [*malB* ^*+*^ ]_K-12_(λ^S^) pLysSRARE[*T7p20 ileX argU thrU tyrU glyT thrT argW metT leuW proL ori*_*p15A*_ ](Cm^R^)) was transformed with pET-21a-*Cpmug* vector and grown in 2xYT medium containing 100 µg/mL of ampicillin, at 37°C overnight under constant stirring (180 rpm). When the culture was grown until the absorbance at 600 nm of 0.6, the temperature was decreased to 25 °C and protein expression was induced with 0.5 mM isopropyl-β-D-thiogalactopyranoside (IPTG) and 1% glucose, followed by incubation for 4 h. Subsequently, they were centrifuged, resuspended in buﬀer (60 mM Tris-HCl, pH 6.8, 10% glycerol, 5% β-mercaptoethanol, 2% SDS, 0.5% bromophenol blue), and subjected to polyacrylamide denaturing electrophoresis gel 12% 29:1 acrylamide/bis-acrylamide (SDS-PAGE) to conﬁrm expression. After that, the cells were resuspended in the binding buffer (10 mL of 20 mM NaH_2_PO_4_, 500 mM NaCl, 40 mM imidazole, 6 M de urea, pH 7.4), lysed by five sonication pulses of 30 sec each resting on ice. 

The soluble fraction was obtained by centrifugation at 10.000 × g for 20 min 4 °C and loaded into a HisTrapTM FF crude (GE Healthcare) column pre-buffered with the binding buffer using a flow at 1 mL/min controlled by a peristaltic pump Miniplus Evolution (Gilson) at 4 °C. The column was washed six times with different solutions (10 mL of 20 mM NaH_2_PO_4_, 500 mM NaCl, 40 mM imidazole, pH 7.4), where the urea concentration deduces 1 M in each solution (6 to 0 M). The elution was performed with 20 mM NaH_2_PO_4_, 500 mM NaCl, 500 mM imidazole, pH 7.4, and 1 mL was recovered for each fraction. A 15% SDS-PAGE analysed the flow-through purity, and the protein concentration was measured by Bradford assay (Bradford, 1976). The pure protein was dialysed in membrane 10 MW (SERVA) 50 mM Tris-HCl, pH 8.0, 50 mM NaCl, 1 mM EDTA.

**Immunodetection of *Cp*Mug**

From a 15% SDS-PAGE assay, the proteins were transferred to a PVDF membrane (Immobilon-MERCK) in a blotting buffer (5 M methanol, 6.25 mM Tris base, 0.048 M glycine, pH 8) using a transfer cassette (Loccus Biotechnology). The membrane was incubated in blocking solution (PBS: 8 mM Na_2_HPO_4_, 2 mM NaH_2_PO_4_, 10 mM NaCl; 0.05% Tween 20, and 10% skimmed milk powder) for 16 h under constant stirring at 4 °C, followed by incubation with anti-histidine monoclonal antibody conjugated with alkaline phosphatase (Sigma-Aldrich) for 2 h under stirring (1:10000 dilution). The membrane was incubated with alkaline phosphatase substrate: 0.02% nitro-blue tetrazolium (NBT) and 0.016% 5-bromo-4-chloro-3-indolyl phosphate (BCIP) (Promega), along with 5 mL of alkaline phosphatase buffer (100 mM Tris-HCl at pH 9.5, 100 mM NaCl, and 5 mM MgCl_2_) until chromogenic detection of bands.

***In vitro* evaluation of uracil DNA glycosylase activity of *Cp*Mug by fluorescence detection**

Two 60-mer annealed oligonucleotides containing a U:G were used as a substrate for the *Cp*Mug activity assay. The U-oligo: 5’-CCCCCGATTTAGAGCTTGACGGGGAACAAGCTTCTCGGCG**U**ACGTGGCGAGAAAGGAGGA-3’ was acquired 5’ end-labelled with 6-carboxyfluorescein (FAM) containing uracil at the 40th position. A 10 µL annealing reaction was run with 100 mM Tris-base, 50 mM NaCl, 10 µM U-oligo, and 12 µM of G-oligo: 5’-TCCTCCTTTCTCGCCACGT**G**CGCCGAGAAGCTTGTTCCCCGTCAAGCTCTAAATCGGGGG-3’. The annealing reactions were conducted at 95 °C for 5 min and cooled at 5 °C each 2 min until room temperature. The oligonucleotide annealing confirmation was analysed in a 20% polyacrylamide gel. The *Cp*Mug activity assay was performed using 19 µM of *Cp*Mug in buffer containing 50 mM Tris-HCl, pH 8.0, 50 mM NaCl, 1 mM EDTA, and 0.03 µM annealed oligonucleotide at 37 °C for 18 h (Catarina et al., 2018). As a positive control, 0.03 µM annealed oligonucleotide was incubated with 2 Units of commercial *E. coli* UDG (Invitrogen). The oligonucleotide in a buffer without enzyme addition was used as a negative control. The resulting AP site was cleaved by hot alkaline treatment with 0.02 M NaOH at 95 °C for 20 min. The DNA fragment analysis was carried out by capillary electrophoresis in an automatic sequencer AB3500 (Life Technologies). Each sample was prepared with 0.8 µL of the enzymatic reaction product, 0.2 µL of 600 LIZ Size Standard (GeneScan), and 9 μL of Hi-Di formamide (Life Technologies). The reaction was heated to 90 °C for 2 min, followed by ice-cooling. The snapshot analysis method was used for fragment analysis, and GeneMapper software was used to read the results. 

***In vitro* evaluation of DNA glycosylase activity of *Cp*Mug in** ^32^ **P labelled lesioned oligos.**

Initially, four different DNA oligonucleotides containing lesions were labelled with [γ- ^32^P]ATP (3×10^3^ Ci/mmol, 20 μCi per 40 pmol of the substrate) using the DNA 5’ End Labeling System kit (Promega): A) 5’-CCGCTAG**U**GGGTACCGAGCTCGAAT-3’ where uracil paired with guanine; B) 5’-CCGCTAGCGGGT**(THF)**ACCGAGCTCGAAT-3’ where the tetrahydrofuran (THF) paired with cytosine; C) 5’- CCGGTGCATGACACTGT**Tg**ACCTATCCTCAGCG -3’ where the Tg paired with adenine, and D) 5’TCACGGGATCAATGTGTTCTTTCAGCTC**8oxo**CGATCTAGACGGAAGGAATACC -3’ where the 8-oxo paired with cytosine. Then, these oligos were hybridised with the non-labelled complement to produce the double strand. It was added 37 nM of recombinant *Cp*Mug to the BER buffer (50 mM Hepes KOH pH 7.8, 0.36% p/v BSA, 70 mM KCl, 5 mM MgCl_2_ and 0.5 mM DTT) and then incubated with 2 pg of hybridised oligonucleotides with or without a subsequent incubation with 1 U Exonuclease III (*E. coli* AP endonuclease, New England Biolabs). As a positive control, the oligo containing uracil was incubated with 1 U of an *E. coli* uracil‐DNA‐glycosylase (New England Biolabs); the oligo containing THF was incubated with 1 U Exonuclease III (*E. coli* AP endonuclease, New England Biolabs); the oligo containing Tg was incubated with 1 U Endonuclease III (*E*. *coli* NTH1, New England Biolabs) and the oligo containing 8-oxo was treated with 1 U of 8-oxoguanine DNA glycosylase (*E.coli* FPG, New England Biolabs). All reactions were run in the BER buffer (50 mM Tris-HCl, pH 8.0, 50 mM NaCl, 1 mM EDTA) for 2 h at 37 ºC. The untreated oligos were used as a negative control. The reactions were inactivated at 75 °C for 10 min, and the addition of one volume of loading buffer (96% v/v formamide, 20 mM EDTA, 5 mM Tris pH 7.5, xylene cyanol 0.05% p/v, bromophenol blue 0.05% p/v). After heating at 95 °C for 5 min, the samples were electrophoretically separated in denaturing 6 M Urea 20% acrylamide gel. Labelled oligos were detected using a phosphorimaging device (BioRad). 

## Results 

**The presence of the *mug* gene is not correlated with pathogenicity in *orynebacterium spp*.**

**Figure 1 -:**
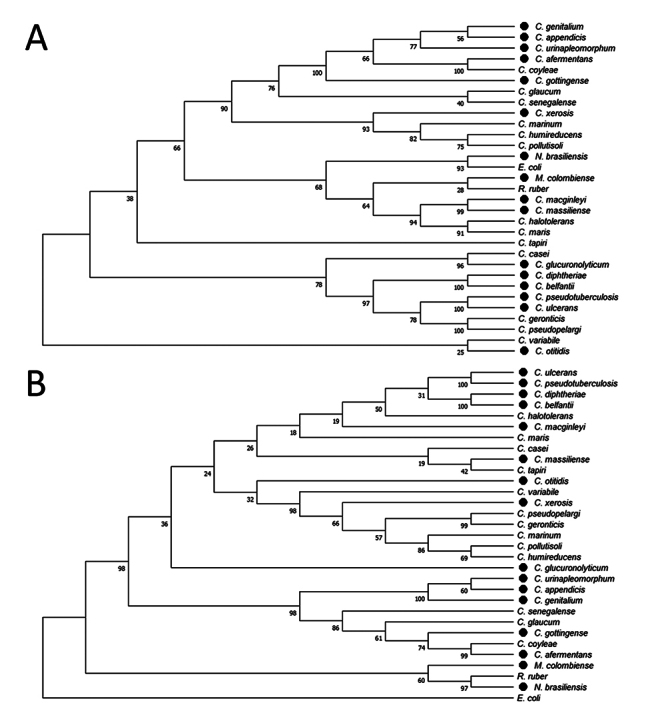
Phylogenetic analysis using the neighbor-joining method: A) The Mug phylogenetic tree was constructed using the sequence of 27 bacteria of *Corynebacterium* spp., species of the CMNR group, and *E. coli*; B) The same cells were used to construct 16S rRNA phylogenetic tree (used as control). The percentages of replicate trees in which the associated taxes clustered together in the bootstrap test (1000 replicates) are shown next to the branches. The evolutionary distances were accurately computed using the Poisson correction method, with the number of amino acid substitutions per site as the unit of measurement. The MEGA X software was employed for the evolutionary analysis. The pathogenic species have been pointed with black circles.

Among the 100 *Corynebacterium* spp. used in this analysis, 55 were pathogenic species, and 45 were non-pathogenic. The mug gene/protein was identified in 27 bacterial genomes, with 14 of these species being pathogenic and 13 non-pathogenic. (Table S1). After conducting a phylogenetic analysis of Mug’s amino acid sequences across 27 Corynebacterium species and CMNR group representatives, a corresponding pattern of clusters was identified when compared to the 16S rRNA phylogenetic tree (Figure 1 A  and B). The similarities between the branches and the bacteria positions among the trees validated this analysis since most *Corynebacterium* species, and CMNR representatives appear in the same branches in both trees. The species *C. macginleyi*, *C. massiliense*, *C. halotolerans* and *C. maris* did not remain in fixed groups, changing their position according to the sequence analysed. The distribution of species within the branches of the Mug tree did not show any correlation with pathogenicity. Both pathogenic and non-pathogenic species were found in the same branch (Figure 1 A ). 

**Catalytic amino acid residues that play an important role in uracil DNA glycosylase enzyme have been identified in *Cp*Mug**

The *Cpmug* gene contains 636 base pairs (GenBank: CP001809.3) and encodes a protein with 211 amino acids (GenBank: ADL20529.1) with a molecular mass of 22.8 kDa, showing a hydrophilic profile (-0.09 hydropathicity index) with an isoelectric point estimated at 6.43. The *Cp*Mug protein sequence showed 86.54% of similarity to Mug from *C. ulcerans* and 70.94% to Mug from *C. diphtheria* (both pathogenic species), 72.00% similarity to Mug from *C. pseudopelargi* and 71.50% to Mug from *C. geronticis* (both non-pathogenic species), 40.1% similarity to Mug from *M. colombiense*, 46.9% to Mug from *R. ruber* and 37.2% to Mug from *N. brasiliensis* (the three CMNR representatives) and 41.6% similarity to *Ec*Mug (data not shown)*.*

**Figure 2 -:**
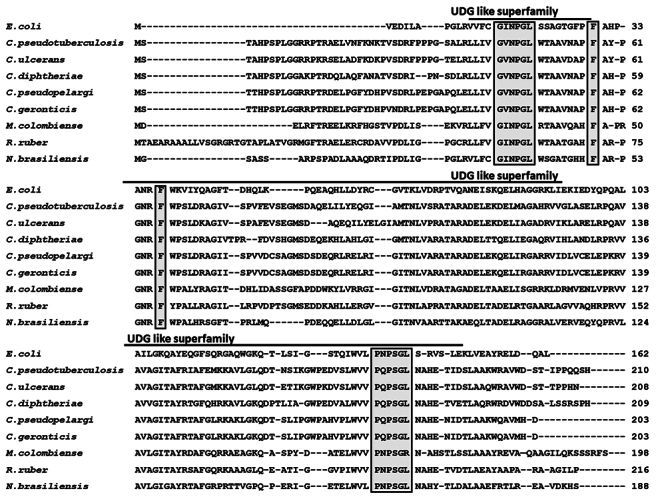
Alignment of Mug protein sequences from different species. The amino acid sequences of *E. coli*, *C. ulcerans*, *C. diphtheria*, *C. pseudopelargi*, *C. geronticis*, *M. colombiense*, *R. ruber,* and *N. brasiliensis* were obtained from GenBank and aligned using T-Coffee software. Black boxes indicate the residues involved in the catalytic activity. Identical amino acid residues appear in grey, while the Uracil-DNA glycosylase-like motif is highlighted and outlined.

When analyzing the catalytic residues associated with Mug activity in *E.coli*, it was observed through alignment that the motifs related to the recognition and processing of uracil, previously described for *Ec*Mug, are also preserved in *Cp*Mug and the other analyzed species (Figure 2) (Barrett et al., 1998). The *Ec*Mug has the G**I**NPGL motif (16 to 21), responsible for the cleavage of the N-glycoside and base excision from the DNA backbone (Barrett *et al*., 1998; (Schormann, Ricciardi and Chattopadhyay, 2014). This motif is preserved in the CMNR group representatives, except for the Corynebacterium genus, where the Iso-17 was exchanged for Val (G**V**NPGL) (Barrett *et al*., 1998). The Phe-30 in *Ec*Mug is responsible for a specific peptide nitrogen interaction with the O4, forming a π-π stacking interaction with uracil, and is conserved in all analysed species (Barrett *et al*., 1998). The Lys-68 of *Ec*Mug is presented in the catalytic pocket and interacts with the oxygen attached to the second carbon in uracil, and this residue was replaced with Asn in all CMNR group representatives. Finally, in *Ec*Mug, the motif P**N**PSGL**S** (139-145), involved in the hydrogen bond interaction with guanine in U=G mismatch through specificity with N1 and N2 of the base, was replaced for P**Q**PSGL**N** in all Corynebacterium. In this motif, the Asn-136 and the Ser-145 in *Ec*Mug were replaced by Gln-179 and Asn-184 in Corynebacterium (Barrett *et al*., 1998). 

**Computational analysis shows that *Cp*Mug exhibits a significant affinity for uracil**

**Figure 3 -:**
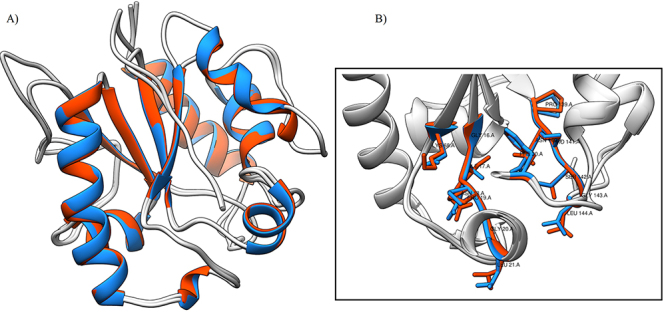
Comparative modelling of *Cp*Mug based on PDB structure of *Ec*Mug (1MWI). A) Structural alignment between the template *Ec*Mug (in red) and model for *Cp*Mug (in blue). B) The catalytic residues are magnified and distinguished. The models were analysed using the UCSF Chimera visualisation software.

To create a model of *Cp*Mug using structural homology, we selected three different *Ec*Mug structures as templates: 1MWI (2.35 Å resolution), 1MWJ (2.85 Å resolution), and 1MTL (2.8 Å resolution). These templates were selected based on their satisfactory e-values (5e-24), high identity (34.97%), and query coverage scores (77%) for *Cp*Mug, as per MEGAX analysis. Additionally, the availability of crystallographic structures helped us determine how they could interact with a DNA molecule containing uracil. To determine the accurate location of the catalytic residues in the *Cp*Mug model, we assessed the top ten models (ranked by DOPE score) (Shen and Sali, 2006) by overlaying them using the UCSF Chimera visualisation software (Figure S1). Following the generation of the protein structure and quality assessment using structural energy and stereochemical features, a three-dimensional model for *Cp*Mug was constructed. Table S2 details the quality verification process and selection of the best structures for developing the *Cp*Mug model. In summary, the selected structure had a DOPE score of -19910, ERRAT score of 87.8, Verify3D of 84.44%, QMean -2.75, Z-values -2.56 and Ramachandran Plot score of 88.2%. The proposed *Cp*Mug structure is composed of six β-strands and four α-helices, according to UCSF Chimera software data; these results show a secondary structure pattern, which resembles that of *Ec*Mug in the organisation, composition, and position. To compare the structures of *Ec*Mug and *Cp*Mug, we created structural alignments and analysed the catalytic residues of both. As shown in Figure 3 A , there were some differences between the two structures, and we found that three catalytic amino acid residues in *Cp*Mug (Ile17-Val45, Lys68-Asn102, Asn140-Gln179) had been replaced, as represented in Figure 3 B . 

To evaluate the binding properties of *Cp*Mug protein with four types of lesions, we analysed the protein-DNA complexes generated by molecular docking. We compared them with the *Ec*Mug template structure. The catalytic residues from *Ec*Mug and *Cp*Mug were used to conduct the molecular docking analyses (Table S3), but only those directly involved in base excision were considered for distance calculation. According to Tables 1 and 2, the distance between the uracil base found in the *Ec*Mug-DNA and *Cp*Mug-DNA complexes was shorter than other lesions. The average distance from the uracil base to *Ec*Mug and *Cp*Mug was 7.459 Å and 7.836 Å, respectively, followed by the distances to the 8-oxo, Tg, and THF lesions in that order (Table S4). Moreover, uracil and 8-oxo showed a similar distance to *Cp*Mug and *Ec*Mug regarding the proximity of residues responsible for guanine interaction with the lesions.

**Table 1 -:** Distance between catalytic residues from *Ec*Mug and DNA lesions*.*

Models	Lesion type	Catalytic residues ^a^								Average
		Gly16	Ile17	Asn18	Pro19	Gly20	Leu21	Phe30	Lys68	
*Ec*Mug	U	9.25	5.81	5.66	7.22	6.38	7.47	6.63	11.26	7.459
*Ec*Mug	8-oxo	9.66	6.65	5.18	7.08	8.8	7.71	6.7	11.54	7.914
*Ec*Mug	Tg	15.69	12.77	8.84	8.89	8.62	5.87	10.93	15.86	10.932
*Ec*Mug	THF	17.08	14.18	10.7	10.7	8.34	7.36	12.29	17.4	12.256

a Distance measured in angstroms between the closest atom in the lesion and catalytic residues concerning the regions described by the base excision function of *E. coli* (16-21; 30; 68 residues).

**Table 2 -:** Distance between catalytic residues from *Cp*Mug and DNA lesions*.*

Models	Lesion type	Catalytic residues ^a^								Average
		Gly44	Val45	Asn46	Pro47	Gly48	Leu49	Phe58	Asn102	
*Cp*Mug	U	9.51	6.96	5.93	7.02	6.4	7.02	7.69	12.17	7.836
*Cp*Mug	8-oxo	10.66	6.84	5.26	7.51	6.86	8.25	8.99	11.55	8.241
*Cp*Mug	Tg	15.29	11.69	9.25	10.05	8.51	7.86	10.49	15.52	11.084
*Cp*Mug	THF	17.9	14.75	11.42	11.09	9.43	7.36	10.93	16.98	12.483

a Distance measured in angstroms between closest atoms in the lesion and catalytic residues concerning the regions described by base excision function of *C. pseudotuberculosis* (44-49; 58; 102 residues).

**Figure 4 -:**
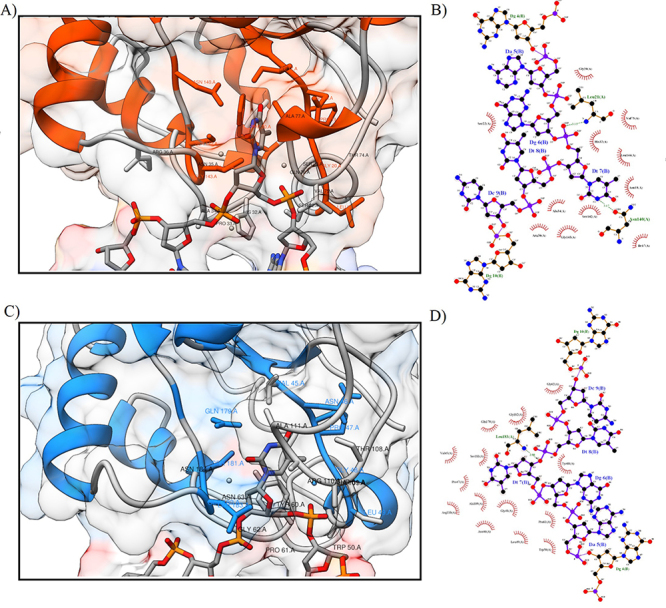
Molecular docking of *Ec*Mug /*Cp*Mug with Uracil. A and B represent *Ec*Mug in complex with uracil, and C and D represent *Cp*Mug in complex with the same lesion. The images were generated by molecular docking calculations and selected after manual assessment. The details of the DNA-binding sites of *Ec*Mug (1MWI) and *Cp*Mug show possible key interacting residues (6.5 Å distance from the lesion). The complexes were analysed using the UCSF Chimera visualisation software.

*Cp*Mug and *Ec*Mug maintained the similarity of the base positions inside the catalytic pocket (Figure 4). *Ec*Mug (Figure 4 A  and B) was used as a positive control to validate the molecular docking results since its complex structure (protein x substrate) was already solved by x-ray diffraction. Figure 4 C  and D display the catalytic residues of *Cp*Mug and its interaction with uracil in the DNA-protein complex. Due to the proximity of residues, the uracil lesion was maintained inside the catalytic pocket. Upon performing a similar analysis on complexes that include lesions such as 8-oxo (Figure S2), Tg (Figure S3), and THF (Figure S4), it was noted that all these lesions were situated outside the catalytic pocket. This was due to their lower affinity towards the catalytic residues.

**The electrostatic potential surface of *Ec*Mug and *Cp*Mug suggests interaction with DNA**

**Figure 5 -:**
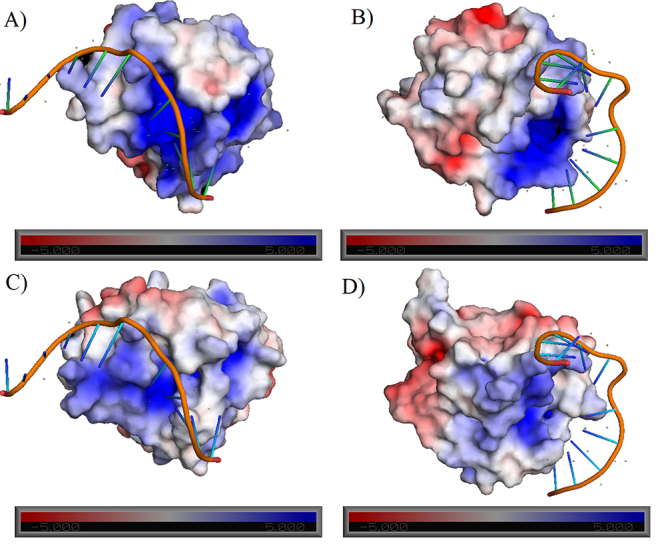
Electrostatic potential surface of *Ec*Mug and *Cp*Mug models in interaction with DNA molecules containing uracil. A-B) Two different spatial visualisations of *Ec*Mug protein surface charges. C-D) Two different spatial visualisations of *Cp*Mug protein surface charges. The DNA molecules are represented in orange. Positive charges are in blue tones, and negative charges are in red, as indicated by the colour grad. The positive (+) and negative (-) signs represent the presence and absence of this compound in the reaction. Positive charges are in blue tones, and negative charges are in red tones, as indicated by the colour gradient bars below each structure. Proteins were structurally aligned, and all molecules had the same spatial orientation as the template. The assignment and visualisation were performed using the APBS tools plugin of PyMOL software.

The DNA-protein complex containing uracil was examined for DNA position in relation to its overall surface charge, using the relative position between DNA and protein molecules in the crystallized complex. Two spatial visualisations of *Ec*Mug protein surface charges are presented in Figure 5 A  and B and for *Cp*Mug in Figure 5 C  and D. Although both have an overall negative charge, *Cp*Mug has a total charge of -5e, while *Ec*Mug has an overall charge of -8e (Figure 5). 

**The purified *Cp*Mug can recognise and remove uracil from DNA**

**Figure 6 -:**
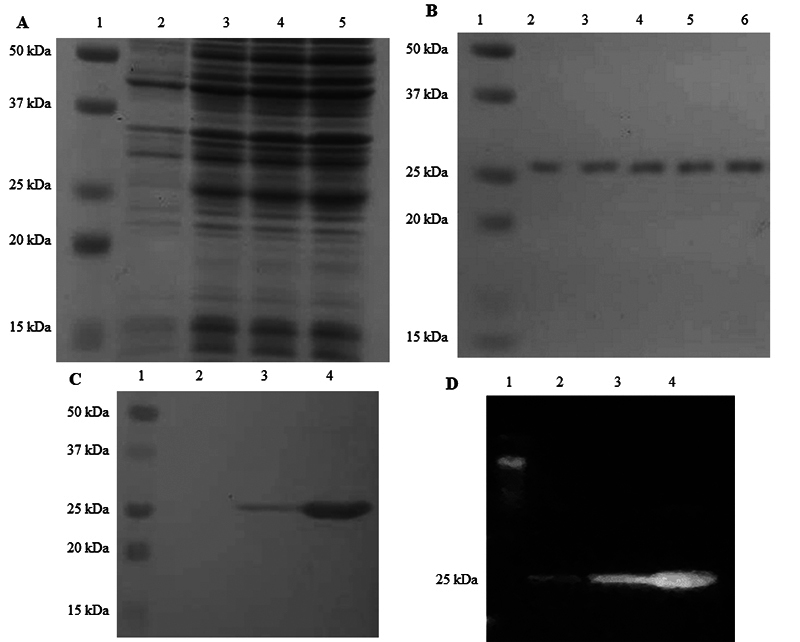
Expression and purification of recombinant *Cp*Mug, confirmed by immunodetection. The samples were subjected to gel electrophoresis in 15% SDS-PAGE stained with Coomassie blue R-250 A) Expression of recombinant protein showing: 1 - Protein standard; 2 - Cellular lysate before IPTG induction; 3 to 5 - Cellular lysate after 2, 4 and 6 h of induction, respectively. B) Recombinant protein purification: 1 - Protein standard; 2 to 6 - Elution samples of purified *Cp*Mug. C) PVDF membrane containing the purified recombinant after performing Ponceau S staining; 1 - Protein standard; 2 to 4 - purified recombinant protein D) Anti-polyhistidine Immunodetection in PVDF membrane containing the purified recombinant; 1 - Protein standard; 2 to 4 - purified recombinant protein.

**Figure 7 -:**
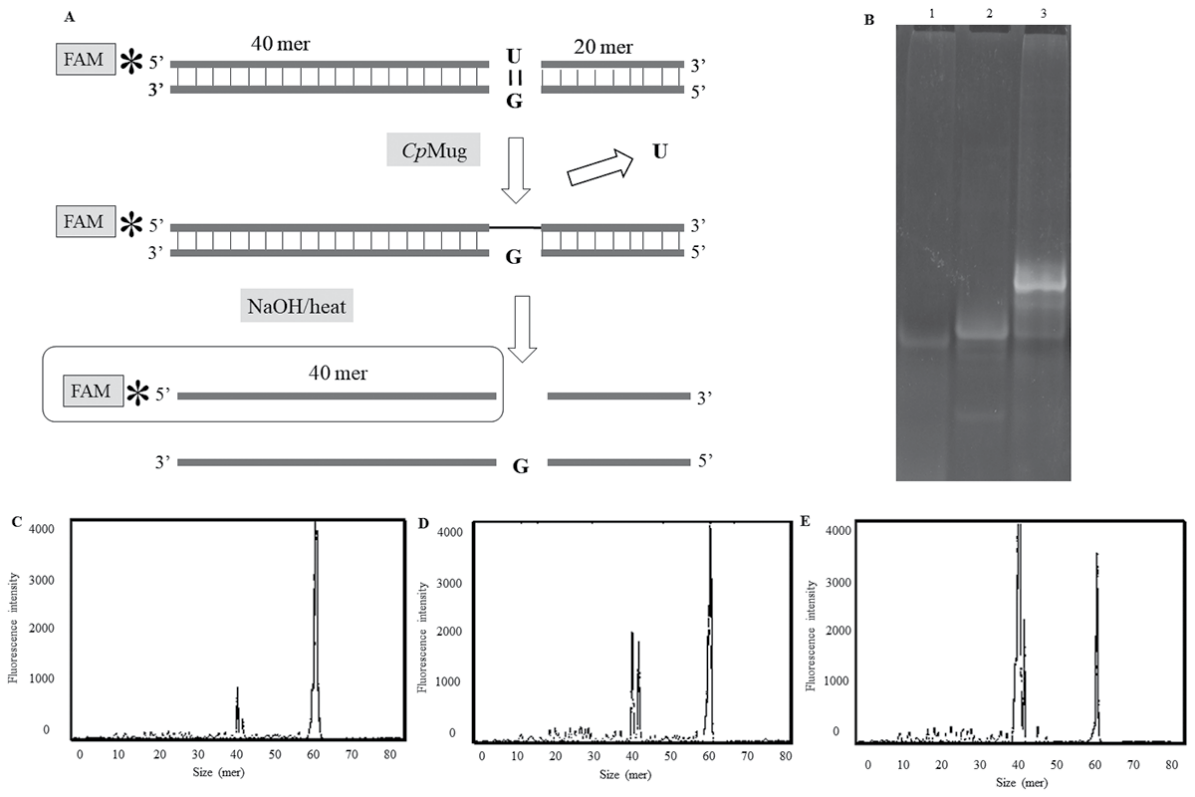
Evaluation of Uracil DNA glycosylase activity of *Cp*Mug. A) Schematic representation of the enzymatic protocol used for 60-bp oligonucleotides labelled with ﬂuorescein at the 5′ position B) 20% polyacrylamide gel stained with ethidium bromide where lane 1 contains a single strand 60-mer oligo with G at 20-mer; lane 2 contains a 60-mer oligo labelled with FAM with U at the 40-th position; lane 3 contains annealed U/G oligos. C) Fragment analyses of the Glycosylases assay negative control with the U/G oligonucleotide treated only with NaOH. D) Positive control with de U/G oligonucleotide treated with commercial UDG from *E. coli* (New England Biolabs). E) *Cp*Mug incubated with the U/G oligonucleotide. Three independent experiments were performed.

The *Cp*Mug recombinant protein was successfully expressed in an *E. coli* model, purified through affinity chromatography (Figure 6 A  and B) and confirmed by immunodetection (Figure 6 C  and D). A DNA glycosylase assay was performed to determine if *Cp*Mug could recognise and remove uracil from the oligonucleotide, as depicted in the schematic representation (Figure 7 A ). Initially, the 60-mer uracil-containing oligo (labelled with 6-FAM at 5’-end) was hybridised with its counterpart oligo. When submitted to electrophoresis, the annealed oligos migrate slower than the unhybridised ones (Figure 7 B  lane 3). After the enzymatic reaction upon the annealed oligos, the reaction products were denatured and solved in a capillary sequencer. In the absence of *Cp*Mug, the negative control reaction displays a peak in the 60-mer size oligonucleotide position (indicating the oligo without cleavage), along with a small amount of 40-mer size oligo produced by spontaneous cleavage (Figure 7 C ). In the positive control reaction, which consists of the oligo and UDG, there are two peaks observed. The first peak is in the size of 40-mer, indicating UDG activity. The second peak is in the size of 60-mer, which is the result of the oligo without cleavage (Figure 7 D ). The *Cp*Mug enzymatic activity resulted in a peak at the 40-mer size position higher than UDG, suggesting its ability to recognise and remove uracil (Figure 7 E ). 

***Cp*Mug can remove uracil from DNA but does not affect THF, 8-oxo, or Tg lesions**

**Figure 8 -:**
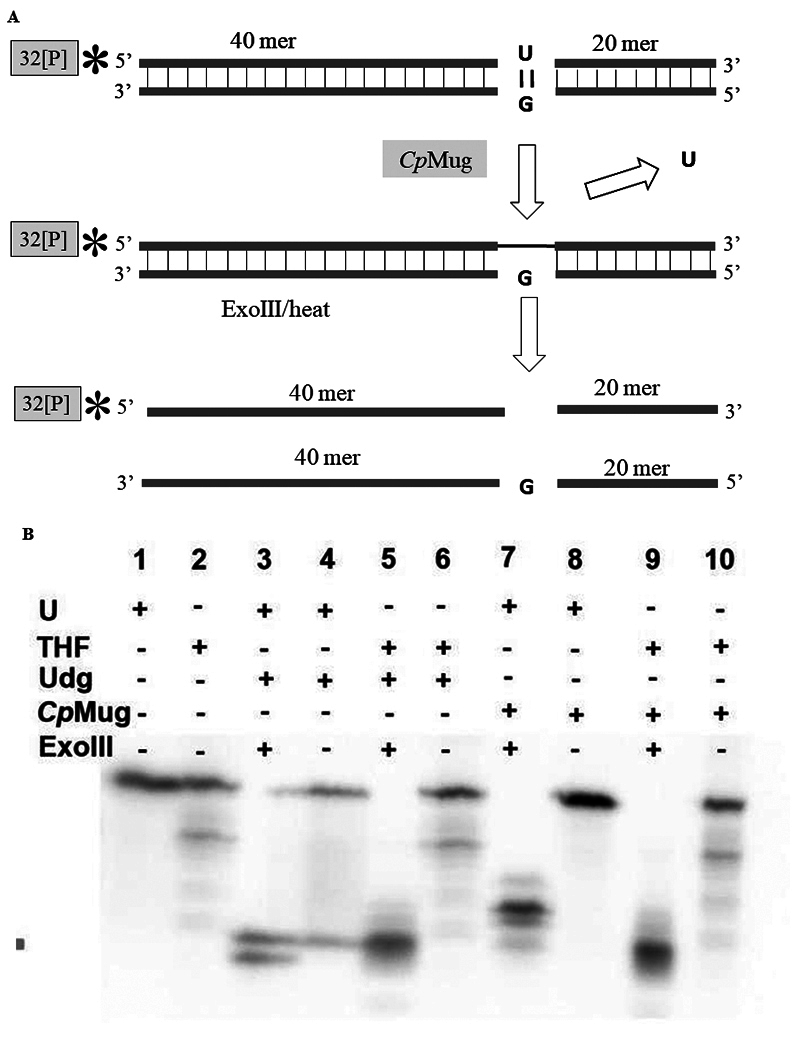
Evaluation of Uracil DNA glycosylase and AP lyase activity of *Cp*Mug. A) Schematic representation of the enzymatic protocol used on oligonucleotides containing a modified base and labelled with ^32^P at the 5’ position. B) Denaturing 6 M Urea- 20% acrylamide gel after enzymatic treatment. The positive (+) and negative (-) signs indicate the presence or absence of each compound in the reaction. U represents the double-strand oligonucleotide containing uracil/guanine, THF is the double-strand oligonucleotide containing the AP site analogue tetrahydrofuran, UDG is the commercial uracil DNA glycosylase from *E. coli*, and *Cp*Mug is the recombinant mismatch uracil DNA glycosylase purified from *C. pseudotuberculosis*. ExoIII is the commercial Exonuclease III that processes the AP-formed site (from *E. coli*).

A distinct method was employed to confirm the uracil DNA glycosylase activity of *Cp*Mug. Additionally, investigations were conducted to verify its effect on other lesions and to establish whether the protein possesses an AP lyase activity. Figure 8 A  shows a schematic representation of the oligonucleotides containing uracil (labelled with ^32^P at 5′-end). The oligonucleotides carrying the uracil and THF damage (AP site analogue) were used as a negative control, remaining intact after denaturation processes (Figure 8 - lines 1 and 2, respectively). As expected for commercial UDG (positive control), the uracil-containing oligo was fully cleaved in the presence of Exonuclease III, responsible for processing the AP site formed after uracil removal by UDG (Figure 8 B  -lane 3). The presence of a lower band when the uracil oligonucleotide was treated with UDG only (without ExoIII) suggests spontaneous cleavage of the formed AP site after its uracil has been removed (Figure 8 B  -lane 4). 

The oligo carrying the THF lesion was cleaved by UDG in the presence of ExoIII (Figure 8 B  -lane 5), but the oligo was not cleaved by UDG alone (Figure 8 B  -lane 6). A lower band suggests total processing of the oligonucleotide available containing uracil by the recombinant *Cp*Mug (Figure 8B - lane 7), but only in the presence of ExoIII, since without ExoIII, no such band was observed, indicating no AP lyase activity (Figure 8 B  - lane 8). The THF oligonucleotide was cleaved in the presence of *Cp*Mug and the exonuclease ExoIII (Figure 8 B  - lane 9), but it was not cleaved in the presence of *Cp*Mug alone (Figure 8 B  - lane 10), showing the absence of AP lyase activity.

**Figure 9 -:**
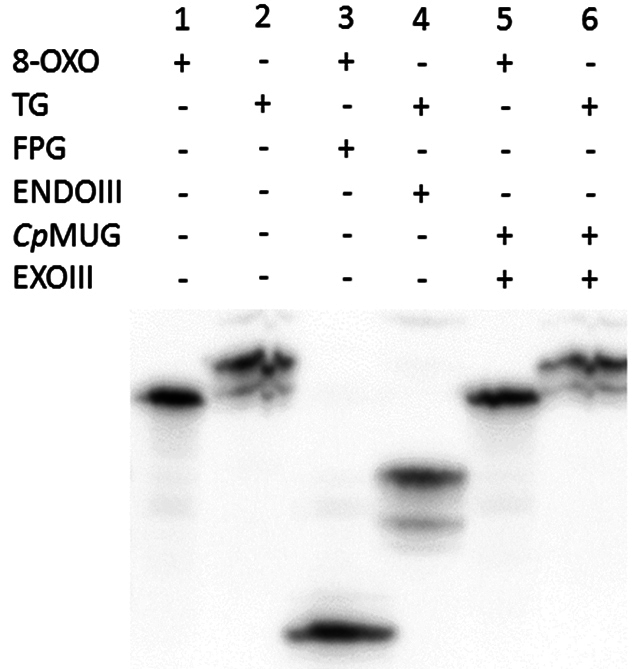
DNA glycosylase assay with [^32^P] labelled oligonucleotides containing 8-oxoguanine and thymine glycol. Denaturing 6 M Urea- 20% acrylamide gel. The positive (+) and negative (-) signs indicate the presence or absence of each compound in the reaction. 8-oxo indicates the oligonucleotide containing 8-oxoguanine; Tg indicates the oligonucleotide containing thymine glycol; FPG indicates the commercial formamidopyrimidine-DNA glycosylase; EndoIII indicates the commercial endonuclease III (from *E. coli*); *Cp*Mug indicates the recombinant mismatch uracil DNA glycosylase purified from *C. pseudotuberculosis*; ExoIII indicates the commercial exonuclease III that processes the AP formed site (from *E. coli*).

To confirm *Cp*Mug’s ability to identify and deal with oxidation products, a follow-up DNA glycosylase assay was conducted using two different oligonucleotides containing 8-oxoguanine or thymine glycol (Figure 9, lanes 1 and 2). The commercial enzymes, FPG and ENDOIII, were able to cleave the 8-oxo and the thymine glycol oligonucleotides, respectively (Figure 9, lanes 3 and 4). When these oligonucleotides were incubated with *Cp*Mug, the absence of a lower band suggests an incapacity of *Cp*Mug to process such oxidative damages (Figure 9, lanes 5 and 6).

## Discussion 

*C. pseudotuberculosis* is a highly resilient and well-adapted microorganism. Being a facultative intracellular pathogen, this organism survives in harsh environments, such as within macrophages often infected during the CLA (Folador et al., 2016; Faria et al., 2016). Specific characteristics, such as low pH, high proteolytic activity, and high oxidative capacity, can damage DNA in this hostile environment. This includes base modifications, the creation of apurinic and apyrimidinic sites (AP sites), and breaks in the DNA strand (Asad et al., 2004; Wozniak and Simmons, 2022). Besides that, *C. pseudotuberculosis* can survive outside of a host for a prolonged period, lasting anywhere from 2 to 8 months. It can withstand various environmental stresses, such as extreme temperatures, dehydration, and exposure to UV radiation (Latif et al., 2015; Catarina et al., 2018). In addition, the genome of *C. pseudotuberculosis* has a high G+C percentage, which makes it more vulnerable to deamination incidents. To survive and protect its genome against various environmental threats and internal adversities, this bacterium has developed an efficient DNA repair system to deal with DNA damage and avoid mutations (Resende et al., 2011; Arantes et al., 2016; Faria *et al*., 2016; Catarina *et al*., 2018).

Studies have indicated that the BER pathway remains intact in the Corynebacterium genus, possessing a comprehensive range of enzymes to deal with DNA oxidative stress and other alterations in nucleotide bases, such as adducts or uracil (Asad et al., 2004; Resende et al., 2011; Arantes et al., 2016; Faria et al., 2016 ). Our previous research revealed that the presence of the mismatch uracil DNA glycosylase (*mug*) gene was limited to pathogenic species (Resende *et al*., 2011). After conducting a comprehensive analysis of all available genomes of Corynebacterium spp., we have discovered that the *mug* gene is present in both pathogenic and non-pathogenic species, as indicated in Table S1. 

Despite the lack of a discernible pattern between the presence of the *mug* gene and pathogenicity in the species examined (Figure 1). It is worth noting that uracil repair mechanisms have been linked to the survival and development of pathogens from the CMNR group (Venkatesh et al., 2003; Kurthkoti and Varshney, 2012; Van and Tang, 2015). 

*Mycobacterium smegmatis*, for example, had its growth in macrophages compromised when the *ung* gene was knocked out (Venkatesh et al., 2003). Furthermore, there was a noticeable synergy in a double mutant of *M. smegmatis* (*ung* and *UdgB*), resulting in heightened sensitivity of the bacteria to RNS and ROS, typically present in the intracellular environment of macrophages (Sassetti and Rubin 2003; Malshetty et al., 2010). In addition, *ung* knockout *M. tuberculosis* also had its survival rate reduced when infecting mice (Sassetti and Rubin, 2003; Kurthkoti and Varshney, 2012).

The genome structure of bacterial species is highly diverse and shaped by various factors, including gene content, duplication, loss, and horizontal gene transfer (HGT) events. To understand the evolutionary history of the *mug* gene, we constructed a phylogenetic tree (Figure 1 A ). Nakamura et al., (2003) showed in their studies that *Corynebacteria* genomes could be helpful in evolutionary biology studies due to the recombinational stability of these species, leading to the following point: the rarity of genome rearrangement indicates that speciation in Corynebacteria has not taken place by genome shuffling, but rather by gene loss (or gain) and nucleotide substitution in the genomes (Hacker and Carniel, 2001). In addition, comparing the G+C content of a gene and the entire genome of an organism can provide clues about the occurrence of horizontal gene transfer; in the case of *C. pseudotuberculosis*, the G+C content of the *Cpmug* gene and the whole genome is similar: 53% *Cpmug* G+C content against 52.2% in the genome (data not shown), suggesting that *Cpmug* was not horizontally transferred but belonged to *C. pseudotuberculosis* genome (Nakamura *et al*., 2003). Since only 27 out of 100 Corynebacteria species appear to have the *mug* gene (Table S2), gene loss probably occurred along the evolution of this genus. Corynebacterium members remained in the same branch of the phylogenetic tree as expected, though four of them grouped close to the former. This result suggests that despite the proximity of the Corynebacterium members, a pattern between the pathogenic and non-pathogenic species is not observed. 

*E. coli*was the first bacterial model to have its mismatch uracil DNA glycosylase identified and characterized (Lindahl et al., 1977). The catalytic amino acid residues of this enzyme were identified, and its crystal structure was solved. In vitro DNA glycosylase assays showed that*EcMug*has the ability to process other types of DNA lesions besides uracil (O’Neill et al., 2003). This protein shared 41.6% similarity with *Cp*Mug and was utilized as a model for comparison through *in silico* analysis (Figures 2 and 3). It has been observed that the G**I**NPGL motif, which plays a crucial role in cleaving the base from the DNA backbone in *Ec*Mug (Schormann et al., 2014), has been conserved in CMNR members, except for those belonging to the Corynebacterium group. These bacteria displayed a substitution of Iso-17 for Val (G**V**NPGL), both non-polar aliphatic amino acids (Figure 2). The Phe-30 is conserved in all analyzed species. In *E.coli*, this residue is inside the catalytic pocket and is responsible for a specific peptide nitrogen interaction with the O4, and base stabilization for remotion of the DNA backbone (Schormann *et al*., 2014). The Lys-68 in *Ec*Mug is another important catalytic residue that is present inside the catalytic pocket and acts by binding and stabilizing the enzyme with the oxygen attached to the second carbon of the uracil promoting the cleavage of the phosphodiester bond 3’ from the abasic site (Barrett et al., 1998). This residue was replaced by Asn in all CMNR members analysed (Figure 2). However, both residues can generate hydrogen bonds with uracil, improving its specificity (Barrett *et al*., 1998). Finally, *Ec*Mug motif P**N**PSGL**S** interacts with guanine in U=G mismatch, contributing to protein flexibility and ensuring the stability of the enzyme/substrate complex for enhanced catalytic efficiency. In the Corynebacterium group, this motif was replaced by P**Q**PSGL**N** (Barrett *et al*., 1998)**.** The Asn-136 and Ser-145 were replaced by Gln and Asn, in which the observed substitutions did not alter the polar properties of the residues (Figure 2). It seems that the representatives of Corynebacterium have identical catalytic residues and motifs. Upon comparison with *E.coli*, it was observed that the substitutions made were conservative and most likely would not impact the Mug function. Furthermore, the high degree of overlap of the catalytic residues in the *Cp*Mug 3D model compared with the solved *Ec*Mug protein complex strongly suggests a preserved functional domain of uracil DNA glycosylase (Figure 3) (Barrett *et al*., 1998; O’Neill *et al*., 2003; Schormann *et al*., 2014).

Computational molecular docking was used to compare the affinity of the *Cp*Mug 3D model for the four different lesions to calculate the proximity of lesion/catalytic residues, validating the *in vitro* analyses. The *in silico* generation of molecular structures from protein sequences using comparative modelling techniques is a helpful strategy, given the restricted number of experimentally solved three-dimensional structures compared with the number of publicly available protein sequences (Bordoli et al., 2009). These computational tools allowed the prediction of the three-dimensional structure and the protein-DNA complex for *Cp*Mug, providing crucial information on the functions of this protein and its putative interaction with DNA lesions at the molecular level. The sequence similarity with the template proteins employed in this study was 41.6%, a result considered significant for proteins with more than 80 residues. This characteristic increases the possibility that the constructed models are structurally similar to the template protein (Santos and Alencastro, 2003).

The Modeller software allowed us to assemble a model using templates to generate a more accurate model for proteins with low coverage. The templates were chosen because they present all the catalytic residues and were crystallised in a complex with a DNA molecule containing uracil. There are three *Ec*Mug structures crystallised in PDB that follow this requirement: 1MWI, 1MWJ, and 1MTL, though there is no crystallised model of *Cp*Mug. The best model created for *Cp*Mug was chosen using the DOPE score of 50,000 models created. This high number of models was generated due to the size difference between the enzyme sequences and the difficulty of obtaining reliable model scores (Table S2). Therefore, it focused on the model’s fidelity in the catalytic regions. The chosen model achieved a high degree of overlap with the ten best models generated (Figure S1). To confirm the overlapping of the catalytic residues in the *Cp*Mug, they were compared with those from *Ec*Mug (1MWI) by structure model superposition (Figure 3). The high degree of superposition of the residues allowed us to analyse the affinity of *Cp*Mug for the lesions tested *in silico*. 

The similarity between *Ec*Mug (Figure 5 A  -B) and *Cp*Mug (Figure 5 C-D) surface charge distributions also validated the *Cp*Mug model through electrostatic profile surface with the Pymol software APBS plugin. Barrett et al., (1998) described a lateral portion of the *Ec*Mug surface, which, although it is not catalytic, is essential for the first contact of the enzyme with the DNA molecule (Barrett *et al*., 1998). This portion is characterised by its concentration of positively charged residues at pH 7. This region was also observed in the *Cp*Mug model, suggesting the importance of this region’s conservation in the uracil mismatch interaction (Figure 5).

Besides uracil, it is known that *Ec*Mug can recognise different kinds of lesions such as thymine mismatched with guanine, 1,N2-ethenoguanine, hypoxanthine, 5-hydroxycitosine and 8-oxoadenine (Talhaoui et al., 2013; Admiraal et al., 2019). We evaluated the *Cp*Mug activity in uracil, thymine glycol, 8-oxoguanine and tetrahydrofuran analogue of the AP site through computational analysis and conducting *in vitro* functional tests. The purified *Cp*Mug was incubated with the annealed oligonucleotides at 37 ºC, a temperature considered optimal for *C. pseudotuberculosis* growth (Hacker and Carniel, 2001). Both glycosylase assays using the purified recombinant *Cp*Mug protein showed uracil glycosylase activity but no AP lyase activity (Figure 7 E  and Figure 8 B ) as previously shown for *Ec*Mug protein, and supported by our *in silico* analysis (Figure 4, Table 1, Table 2, and Figure S2.) (O’ Neill *et al*., 2003). Curiously, although it was known that *E. coli* UDG has monofunctional activity as a DNA glycosylase (Talhaoui *et al*., 2013), a background was observed related to spontaneous AP site cleavage even in the absence of ExoIII (Figure 8 B  - lane 2 and 10). This reveals the instability of the formed AP site, which can undergo spontaneous cleavage after its generation during the removal of uracil by the UDG. Interestingly, this spontaneous cleavage of the AP site after the glycosylase reaction was not observed in the recombinant *Cp*Mug incubation since the oligonucleotide product appears uncleaved (Figure 8 B  - lane 8). According to a study conducted by Srinath et al. (2007),*M.* *tuberculosis*has an enzyme known as UdgB, which can remove uracil from single-stranded DNA. This particular DNA glycosylase also provides protection to the DNA from the harmful effects of the AP sites formed, avoiding the potential spontaneous cleavage that could happen subsequent to the removal of uracil (Baute and Depicker, 2008). Based on this observation, we suggest that a similar kind of protection could be happening in C.*pseudotuberculosis*as well, considering the absence of any lower band (as seen in Figure 8 B  - lane 8).

Since *C. pseudotuberculosis* lives in an intracellular environment and is exposed to high concentrations of ROS/RNS, the importance of DNA glycosylases, such as MutM/Fpg, MutY and here, Mug, has been demonstrated to a better understanding of how *C. pseudotuberculosis* manages to maintain its genomic stability (Baute and Depicker, 2008; Arantes et al., 2016; Faria et al., 2016). To examine the action of *Cp*Mug on oxidative DNA stress, assays were made with two common oxidised base modifications: 8-oxo and Tg and no glycosylase activity was observed in these damages (Figure 9 - lines 5 and 6). This data corroborates with *in silico* observations, where Tg lesion docking analysis failed to generate any protein-DNA complex conformation in which the lesions are correctly oriented at the enzyme active site, demonstrating a repulsion so intense that these lesions would be flipped out of the catalytic pocket (a similar profile was observed for THF) (Tables 1 and 2). Interestingly, the DNA-protein complexes containing the lesion with 8-oxo showed similar proximity to those observed with the uracil lesion (Tables 1 and 2). However, the amino acids that interact with the lesions are not the catalytic ones, and this interaction is due to the main Van der Waals energy (Table S3 and Figure 2). In *C. pseudotuberculosis*, oxidative stress seems to be resolved mainly through the GO system, a BER pathway that can remove 8-oxoguanine to prevent mutations after replication events and can remove the oxidised nucleotides from the nucleotide pool and to avoid misincorporation of this deoxynucleotide in the DNA (Arantes *et al*., 2016; Faria *et al*., 2016). On the other hand, oxidative lesions can distort the helix form of the DNA molecule, corrected by the Nucleotide Excision Repair (NER) pathway, which uses UvrABC proteins for recognition and excision of approximately 15 nucleotides flanking the lesion (Lee and Kang, 2019). Since *C. pseudotuberculosis* has a conserved and active NER pathway, these proteins could also be acting to prevent the harmful effects of oxidised DNA (Catarina et al., 2018; Resende et al., 2020).

Our analysis using computer simulation strongly supports the findings of the *in vitro* tests, confirming the involvement of *Cp*Mug in DNA repair and its specificity for uracil removal. This study provides valuable insights for DNA Repair research in *Corynebacterium pseudotuberculosis,* a pathogen with a significant global economic impact. The findings from this study serve as a basis for future research that aims to discover potential targets for the virulence and pathogenicity of this bacteria.

## Supplementary material

The following online material is available for this article:

Table S1 - List of bacteria species used in the study.

Table S2 - Evaluation of *Cp*Mug after the optimisation procedure.

Table S3 - Evaluation scores of *Cp*Mug and 1MWI after docking lesions.

Table S4 - Distance evaluation in Angstroms scale between Mug (*Ec*Mug or *Cp*Mug) and lesions.

Figure S1 - Superimposed representative *Cp*Mug structures obtained from the ten best models generated by Modeller.

Figure S2 - Molecular docking of *Cp*Mug and interaction with the 8-oxoguanine lesion.

Figure S3 - Molecular docking of *Cp*Mug and interaction with Thymine glycol lesion.

Figure S4 - Molecular docking of *Cp*Mug and interaction with AP site analogue THF lesion.
