# Supplementary material for: Mismatch uracil DNA glycosylase (Mug) is maintained in the Corynebacterium pseudotuberculosis genome and exhibits affinity for uracil but not other types of damage
Source: Genet Mol Biol. 2025 Apr 14;48(2):e20230353. doi: 10.1590/1678-4685-GMB-2023-0353 (PMC12001322; doi:10.1590/1678-4685-GMB-2023-0353)
Supplement: Table S1 - [file 1415-4757-GMB-48-02-e20230353-s1.pdf]

Genetics and Molecular Biology
https://doi.org/10.1590/1678-4685-GMB-2023-0353

Supplementary Material to "Mismatch uracil DNA glycosylase (Mug) is maintained in the Corynebacterium pseudotuberculosis genome and exhibits affinity for uracil but not other types of damage."

Table S1 – List of bacteria species used in the study, contemplating genome size (Mb) and presence of the *mug* gene.

Specie	Access code	Genome (Mb)	Mug	Specie	Access code	Genome (Mb)	Mug
C. accolens	ACGD00000000.1	2.46	-	***C. doosanense***	CP006764.1	2.67	-
C. afermentans	GCA_900156035.1 WP_063937359.1	2.32	+	***C. durum***	GCA_001557475.1	3.40	-
C. ammoniagenes	CP009244.1	2.79	-	***C. efficiens***	GCA_000011305.1	3.20	-
C. amycolatum	ABZU00000000.1	2.51	-	***C. endometrii***	CP039247.1	2.47	-
C. appendicis	GCA_900156665.1 WP_084560468.1	2.24	+	***C.epidermidicanis***	CP011541.1	2.69	-
C. aquilae	CP009245.1	2.92	-	***C. falsenii***	CP007156.1	2.38	-
C. argentoratense	CP006365.1	2.02	-	***C. flavescens***	CP009246.1	2.67	-
C. atypicum	GCA_000732945.1	2.35	-	***C.frankenforstense***	CP009247.1	2.60	-
C. auriscanis	GCA_000767255.1	2.56	-	***C. freiburgense***	GCA_000428805.1	2.91	-
C. aurimucosum	CP001601.1	2.73	-	***C. freneyi***	GCA_000758965.1	3.04	-
C. belfantii	GCA_900205605.1 WP_088266860.1	2.39	+	***C. genitalium***	CM000961.1 WP_005290455.1	2.33	+
C.bouchesdurhonense	GCA_900078305.2	2.25	-	***C. geronticis***	GCA_003813985.1 WP_123933466.1	2.26	+
C. bovis	AENJ00000000.1	2.52	-	***C. glaucum***	CP019688.1 WP_095659816.1	2.51	+

Specie	Access code	Genome (Mb)	Mug	Specie	Access code	Genome (Mb)	Mug
C. callunae	GCA_000344785.1	2.90	-	***C.glucuronolyticum***	GCA_900176155.1 WP_070741595.1	2.82	+
C. camporealensis	GCA_000980815.1	2.45	-	***C. glutamicum***	CP025533.1	3.27	-
C. canis	CP004350.1	2.96	-	***C. glyciniphilum***	CP006842.1	3.57	-
C. capitovis	AQUV00000000.1	1.96	-	***C. godavarianum***	GCA_007559235.1	2.52	-
C. casei	CP004350.1 WP_025387784.1	3.11	+	***C. gottingense***	GCA_003693265.1 WP_095553070.1	2.62	+
C. caspium	ARBM00000000.1	1.84	-	***C. hadale***	GCA_002285075.1	2.57	-
. ciconiae	AQUW00000000.1	2.54	-	***C. halotolerans***	CP003697.1 WP_015400496.1	3.20	+
C. coyleae	GCA_900105505.1 WP_101740223.1	2.48	+	***C. heidelbergense***	GCA_003285565.1	2.29	-
C. crenatum	GCA_000380545.1	3.34	-	***C. humireducens***	CP005286.1 WP_052437776.1	2.65	+
C. cystitidis	FOGQ00000000.1	2.98	-	***C. jeikeium***	CR931997.1	2.41	-
C. dentalis	GCA_900232865.1	2.30	-	***C. kefirresidentii***	GCA_002154655.1	2.62	-
C. deserti	CP009220.1	3.03	-	***C. kutscheri***	CP011312.1	2.37	-
C. diphtheriae	GCA_001457455.1 WP_014318830.1	*2.44*	+	***C. lactis***	CP006841.1	2.66	-
C. lipophiloflavum	ACHJ00000000.1	2.38	-	***C.pyruviciproducens***	GCA_002847965.1	2.79	-
C. lowii	GCA_001412085.1	2.35	-	***C. renale***	GCA_900478035.1	2.33	-
C. lubricantis	GCA_000379425.1	2.94	-	***C. resistens***	CP002857.1	2.60	-
C. macginleyi	GCA_003688935.1 WP_121911730.1	2.41	+	***C. riegelii***	GCA_001263755.1	2.53	-
C. marinum	CP007790.1 WP_042621265.1	2.72	+	***C. sanguinis***	GCA_007641235.1	2.32	-
C. maris	CP003924.1 WP_020934425.1	2.83	+	***C. segmentosum***	GCA_900637825.1	2.37	-
C. massiliense	ATVG00000000.1 WP_022862571.1	2.18	+	***C. senegalense***	GCA_900411315.1 WP_115684769.1	2.31	+
C. mastidis	GCA_002835695.1	2.31	-	***C. simulans***	CP014634.1	2.65	-
C. matruchotii	ACSH00000000.2	2.85	-	***C. singulare***	GCA_007666555.1	2.85	-

Specie	Access code	Genome (Mb)	Mug	Specie	Access code	Genome (Mb)	Mug
C. minutissimum	LS483460.1	2.69	-	***C. sputi***	GCA_000427865.1	2.91	-
C. mustelae	GCA_001020985.1	3.47	-	***C. sphenisci***	GCA_001941505.1	2.59	-
C. mycetoides	LT629700.1	2.34	-	***C. spheniscorum***	GCA_008693095.1	2.45	-
C. nuruki	CP042429.1	3.10	-	***C. stationis***	GCA_001941345.1	2.85	-
C. oculi	GCA_001412105.1	2.41	-	***C. striatum***	CP021252.1	2.85	-
C. otitidis	GCA_000296405.1 WP_004601640.1	2.14	+	***C. tapiri***	GCA_006334925.1 WP_139465212.1	2.25	+
C. pacaense	GCA_900169525.1	3.03	-	***C. terpenotabidum***	CP003696.1	2.75	-
C. pilosum	AREP00000000.1	2.54	-	***C. ulcerans***	CP002791.1 WP_029975220.1	2.49	+
C. pollutisoli	GCA_900177745.1 WP_085549768.1	2.54	+	***C. ulceribovis***	GCA_000372445.1	2.30	-
C. propinquum	GCA_000375525.1	2.55	-	***C. urealyticum***	AM942444.1	2.36	-
C. provencense	GCA_003209395.1	3.08	-	***C. ureicelerivorans***	GCA_000747315.1	2.32	-
C. pseudodiphtheriticum	JIAH00000000.1	2.32	-	***C. urinapleomorphum***	GCA_900155535.1 WP_087116800.1	2.26	+
C. pseudogenitalium	ABYQ00000000.2	2.60	-	***C. variabile***	CP002917.1 AEK37247.1	3.18	+
C. pseudopelargi	GCA_003814005.1 WP_123960591.1	2.34	+	***C. vitaeruminis***	CP004353.1	2.71	-
C. pseudotuberculosis	NC_017301.1 WP_013241493.1	2.33	+	***C. xerosis*** WP-155868633.1	GCA_009730475.1	2.78	+
E.coli	U00096.3 NP_417540.1	5.13	+	***N. brasiliensis***	CP003876.1 WP_042260379.1	8.93	+
M. colombiense	CP020821.1 RAV10536.1	5.62	+	***R. ruber***	CP023714.1 WP_102030849.1	5.57	+
