# Supplementary material for: Mismatch uracil DNA glycosylase (Mug) is maintained in the Corynebacterium pseudotuberculosis genome and exhibits affinity for uracil but not other types of damage
Source: Genet Mol Biol. 2025 Apr 14;48(2):e20230353. doi: 10.1590/1678-4685-GMB-2023-0353 (PMC12001322; doi:10.1590/1678-4685-GMB-2023-0353)
Supplement: Table S2 - [file 1415-4757-GMB-48-02-e20230353-s2.pdf]

Genetics and Molecular Biology
https://doi.org/10.1590/1678-4685-GMB-2023-0353

Supplementary Material to "Mismatch uracil DNA glycosylase (Mug) is maintained in the Corynebacterium pseudotuberculosis genome and exhibits affinity for uracil but not other types of damage."

Table S2 – Evaluation of *Cp*Mug after the optimization procedure.

Models	DOPE score[a]	ERRAT[b]	Verify[c]	QMean[d]	Z-score[e]	Ramachandran Plot[f]			
						R1	R2	R3	R4
***Cp*Mug**	19910	87.8	84.44%	-2.75	-2.56	88.2 %	9.2 %	1.3 %	1.3 %

[a]More negative DOPE (Discrete Optimized Protein Energy) score values tend to correlate with more native-like models. DOPE was generated through many iterations by MODELLER script.

[b]ERRAT score over 80 displays that only a few residues have an elevated error function (error > 95% confidence limit) compared to similar experimental structures. ERRAT was calculated by the SAVES server of UCLA-DOE Lab (http://servicesn.mbi.ucla.edu/SAVES/).

[c]Verify3D result from over 80% displays that the amino acids have compatibility between the 3D model and the amino acid sequence. Verify3D was calculated by the SAVES server of UCLA-DOE Lab (http://servicesn.mbi.ucla.edu/SAVES/).

[d]QMean scores are numerical values close to zero that indicate the geometrical properties (both global and local) are similar to what one would expect from experimental structures of the same size. QMean values were obtained from the https://swissmodel.expasy.org/assess server.

[e]Z-score is used to test if the knowledge-based potentials could recognize a native fold found in experimental structures from other alternatives. The Z-score for these structures had to be within the acceptable range of −12 to 12. The Z-score was calculated by the ProSAWeb server (https://prosa.services.came.sbg.ac.at/).

[f]R1: Residues in most favored regions; R2: Residues in additional allowed regions; R3: Residues in generously allowed regions; R4: Residues in disallowed regions.
