# Supplementary material for: Mismatch uracil DNA glycosylase (Mug) is maintained in the Corynebacterium pseudotuberculosis genome and exhibits affinity for uracil but not other types of damage
Source: Genet Mol Biol. 2025 Apr 14;48(2):e20230353. doi: 10.1590/1678-4685-GMB-2023-0353 (PMC12001322; doi:10.1590/1678-4685-GMB-2023-0353)
Supplement: Table S3 - [file 1415-4757-GMB-48-02-e20230353-s3.pdf]

Genetics and Molecular Biology

https://doi.org/10.1590/1678-4685-GMB-2023-0353

Supplementary Material to "Mismatch uracil DNA glycosylase (Mug) is maintained in the Corynebacterium pseudotuberculosis genome and exhibits affinity for uracil but not other types of damage."

Table S3 – Evaluation scores of *Cp*Mug and 1MWI after docking lesions.

Models	Lesion type[a]	Lesion PDB[b]	HADDOCK score[c]	RMSD[d]	Cluster size	Van der Waals energy	Electrostatic energy	Desolvation energy	Restraints violation energy	Buried Surface Area	Z-score[e]
***Ec*Mug**	U	**1MWI**	-72.6 +/- 1.4	1.0 +/- 0.8	199	-57.2 +/- 2.6	-81.4 +/- 17.7	-0.6 +/- 0.6	15.8 +/- 13.36	1122.8 +/- 38.6	0.0
***Ec*Mug**	8-oxo	**1R2Y**	-75.0 +/- 1.7	0.7 +/- 0.5	199	-64.5 +/- 1.7	-67.5 +/- 9.6	1.0 +/- 0.3	20.5 +/- 20.34	1165.9 +/- 9.2	0.0
***Ec*Mug**	Tg	**2DY4**	-55.7 +/- 5.9	19.2 +/- 1.5	116	-48.1 +/- 3.7	-106.6 +/- 37.3	2.8 +/- 3.5	109.3 +/- 30.48	1013.6 +/- 49.0	-1.4
***Ec*Mug**	THF	**5J5G**	-57.6 +/- 1.3	10.6 +/- 0.2	169	-44.7 +/- 2.3	-131.3 +/- 20.3	1.7 +/- 1.9	117.1 +/- 14.28	926.5 +/- 45.3	-1.0
***Cp*Mug**	U	**1MWI**	-67.8 +/- 7.1	2.0 +/- 1.2	181	-54.6 +/- 4.4	-73.1 +/- 10.2	-2.0 +/- 3.1	34.4 +/- 22.51	1027.5 +/- 47.6	-1.0
***Cp*Mug**	8-oxo	**1R2Y**	-56.2 +/- 4.4	1.1 +/- 1.0	198	-56.6 +/- 6.2	6.8 +/- 23.3	-6.4 +/- 2.4	54.4 +/- 19.80	1061.2 +/- 67.2	0.0

Models	Lesion type[a]	Lesion PDB[b]	HADDOCK score[c]	RMSD[d]	Cluster size	Van der Waals energy	Electrostatic energy	Desolvation energy	Restraints violation energy	Buried Surface Area	Z-score[e]
Cp**Mug**	Tg	**2DY4**	-56.9 +/- 3.9	1.9 +/- 2.2	95	-55.5 +/- 1.7	-63.6 +/- 14.6	-6.5 +/- 2.1	178.4 +/- 22.06	1053.9 +/- 61.7	-1.3
Cp**Mug**	THF	**5J5G**	-65.3 +/- 0.7	0.9 +/- 0.5	176	-52.9 +/- 1.6	-89.8 +/- 7.0	-6.7 +/- 1.8	122.6 +/- 4.00	945.8 +/- 34.8	-1.4

[a]Single strand of DNA containing lesions: U, uracil (5′-CGCGAGUTCGCG-3′); 8-oxoguanine (5′-GTCCA(8OG)GTCTACC-3'); TG, thymine glycol (5'-CG(TG)GGAATGACAGCCGCG-3'); THF, AP site analogue tetrahydrofuran (5’-CCGCTAGCGGGT(THF)ACCGAGCTCGAAT-3’).

[b]Single strand of DNA containing lesions was extracted from crystallographic complexes from Protein Data Bank (PDB) (http://www.rcsb.org).

[c]More negative HADDOCK scores values tend to correlate with best complex docking.

[d]Root Mean Square deviation is relevant when you want to assess the balance and stability of the system.

[e]Z-score indicates how many standard deviations from the average this cluster is located in terms of score (the more negative, the better).
