# Supplementary material for: Mismatch uracil DNA glycosylase (Mug) is maintained in the Corynebacterium pseudotuberculosis genome and exhibits affinity for uracil but not other types of damage
Source: Genet Mol Biol. 2025 Apr 14;48(2):e20230353. doi: 10.1590/1678-4685-GMB-2023-0353 (PMC12001322; doi:10.1590/1678-4685-GMB-2023-0353)
Supplement: Table S4 - [file 1415-4757-GMB-48-02-e20230353-s4.pdf]

Genetics and Molecular Biology
https://doi.org/10.1590/1678-4685-GMB-2023-0353

Supplementary Material to "Mismatch uracil DNA glycosylase (Mug) is maintained in the Corynebacterium pseudotuberculosis genome and exhibits affinity for uracil but not other types of damage."

Table S4 – Distance evaluation in Angstroms scale between Mug (*Ec*Mug or *Cp*Mug) and lesions.

**Models**	**Lesion type**	**Catalytic residues[a]**						**Average**
		**Pro139**	**Asn140**	**Pro 141**	**Ser142**	**Gly143**	**Leu144**	
***Ec*Mug**	U	10.75	4.8	7.58	3.99	4.87	2.63	5.77
***Ec*Mug**	8-oxo	10.32	4.48	6.84	3.34	4.87	5.18	5.84
***Ec*Mug**	Tg	16.15	10.58	11.09	6.76	9.6	6.95	10.19
***Ec*Mug**	THF	17.01	11.77	11.52	7.53	8.1	5.16	10.18
**Models**	**Lesion type**	**Catalytic residues[b]**						**Average**
		**Pro 178**	**Gln179**	**Pro 180**	**Ser181**	**Gly182**	**Leu183**	
***Cp*Mug**	U	11.48	4.2	8.01	3.77	5.14	4.59	6.20
***Cp*Mug**	8-oxo	10.58	5.44	7.55	3.16	4.97	4.87	6.10
***Cp*Mug**	Tg	13.72	9.04	8.21	4.32	4.25	4.66	7.37
***Cp*Mug**	THF	17.58	12.43	11.79	10.86	8.01	8.16	11.47

[a]Distance measured in angstroms between the closest atom from the lesion and catalytic residues concerning the regions described by responsible for guanine interaction of the *E. coli* (139-145 residues).

[b]Distance measured in angstroms between the closest atom from the lesion and catalytic residues concerning the regions responsible for guanine interaction of the C. *pseudotuberculosis* (178-184 residues).

*Residues closest to the lesion when compared to the uracil lesion.
