# Supplementary material for: Mismatch uracil DNA glycosylase (Mug) is maintained in the Corynebacterium pseudotuberculosis genome and exhibits affinity for uracil but not other types of damage
Source: Genet Mol Biol. 2025 Apr 14;48(2):e20230353. doi: 10.1590/1678-4685-GMB-2023-0353 (PMC12001322; doi:10.1590/1678-4685-GMB-2023-0353)
Supplement: Figure S1 - [file 1415-4757-GMB-48-02-e20230353-s5.pdf]

Genetics and Molecular Biology
https://doi.org/10.1590/1678-4685-GMB-2023-0353

Supplementary Material to "Mismatch uracil DNA glycosylase (Mug) is maintained in the Corynebacterium pseudotuberculosis genome and exhibits affinity for uracil but not other types of damage."

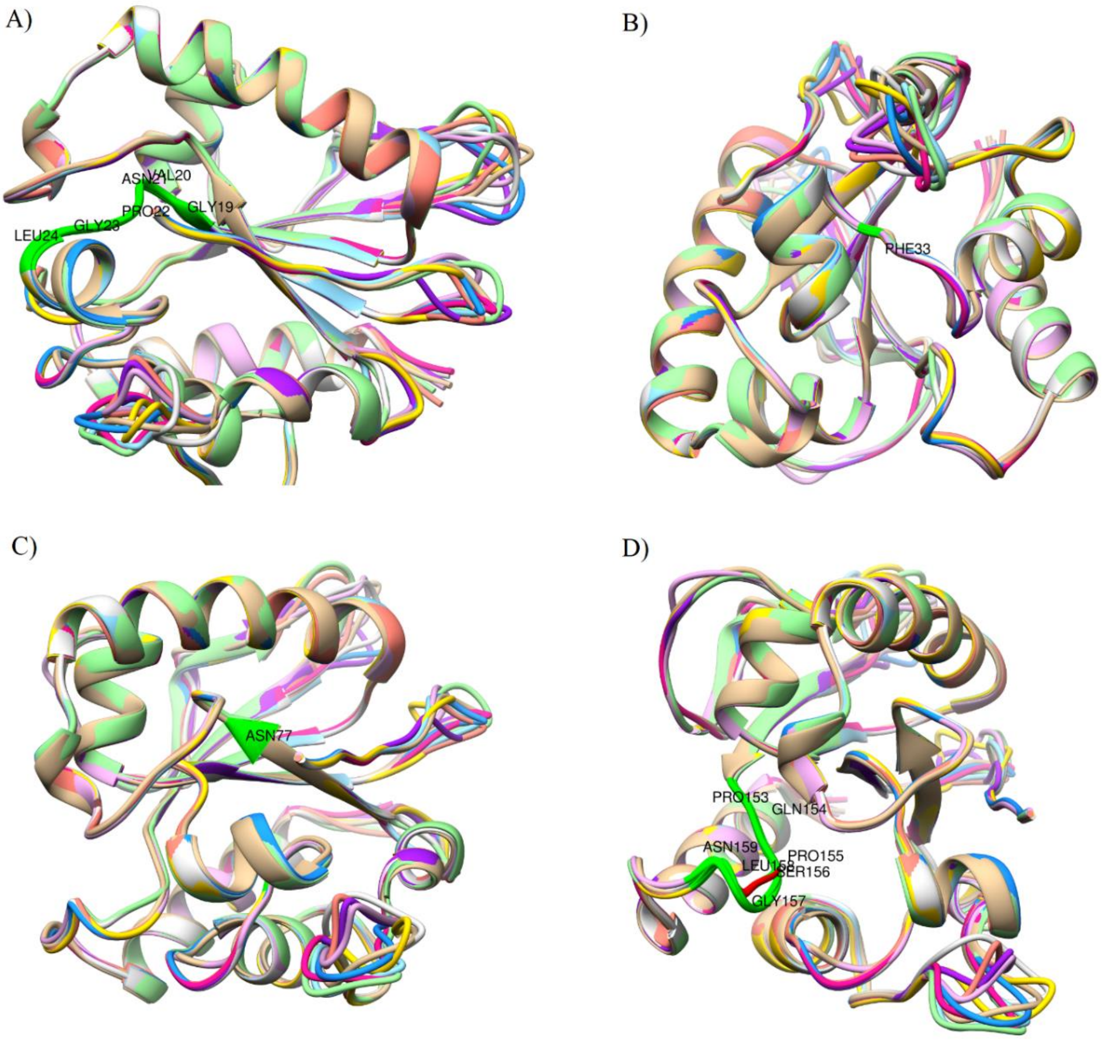

Figure S1 – Superimposed representative CpMug structures obtained from the ten best models generated by Modeller. A, B, C) Structures highlighted in green showing catalytic residues concerning the base excision function regions. D) Structures high lighting in green showing catalytic residues concerning the responsible for guanine interaction regions. Three-dimensional representations were built with the UCSF Chimera.
