# Supplementary material for: Mismatch uracil DNA glycosylase (Mug) is maintained in the Corynebacterium pseudotuberculosis genome and exhibits affinity for uracil but not other types of damage
Source: Genet Mol Biol. 2025 Apr 14;48(2):e20230353. doi: 10.1590/1678-4685-GMB-2023-0353 (PMC12001322; doi:10.1590/1678-4685-GMB-2023-0353)
Supplement: Figure S2 - [file 1415-4757-GMB-48-02-e20230353-s6.pdf]

Genetics and Molecular Biology
https://doi.org/10.1590/1678-4685-GMB-2023-0353

Supplementary Material to "Mismatch uracil DNA glycosylase (Mug) is maintained in the Corynebacterium pseudotuberculosis genome and exhibits affinity for uracil but not other types of damage."

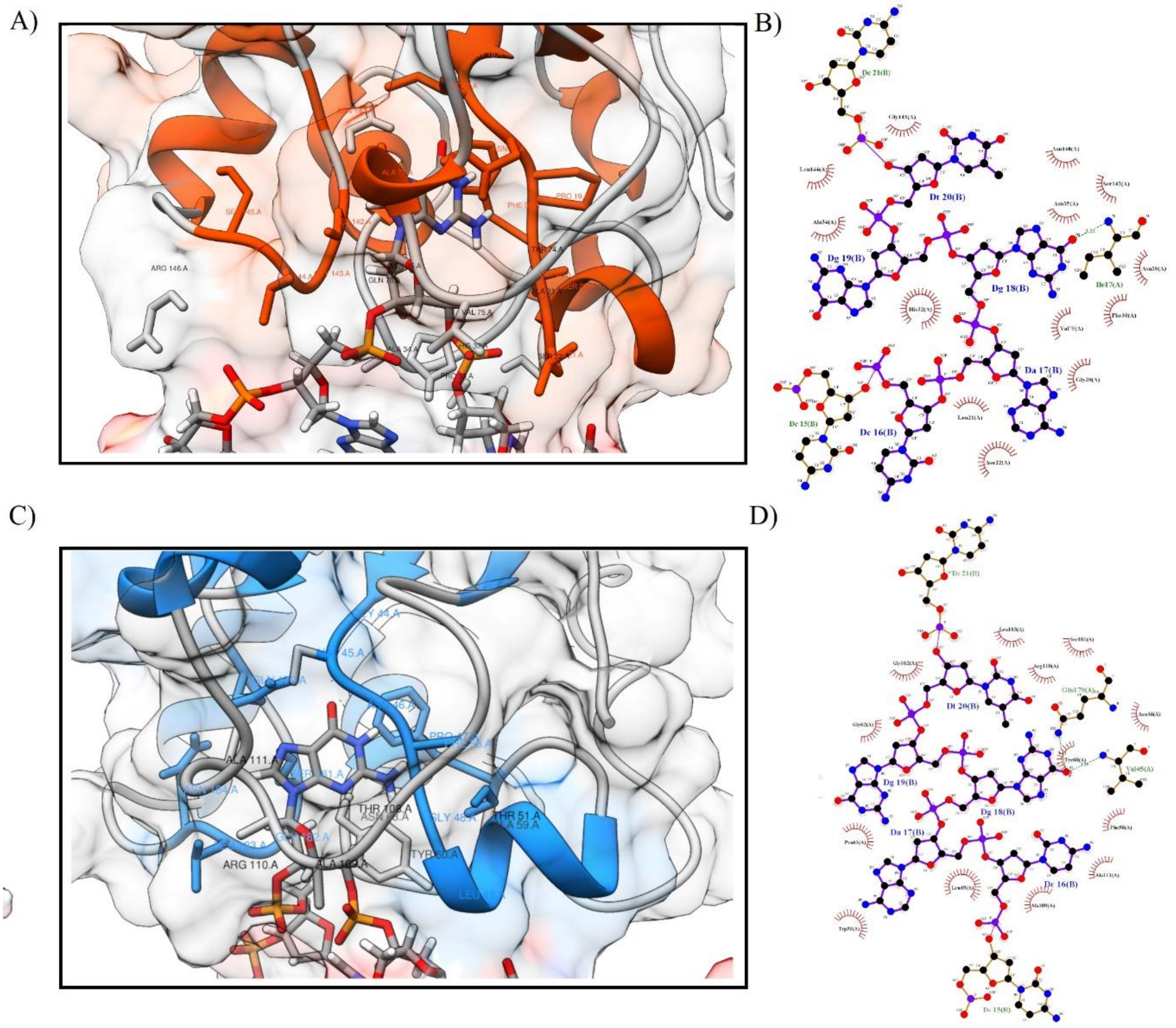

Figure S2 – Molecular docking of CpMug and interaction with the 8-oxoguanine lesion. A) The EcMug (in red) and CpMug (in blue) in the complex with the 8-oxoguanine lesion (in red) were generated by molecular docking calculations and selected after manual assessment. B) Visualization of the amino acid residues that interact with the lesion. The details of the DNA-binding sites of EcMug-1MWI and CpMug show possible key interacting residues (6.5 Å distance from all the lesions). The complexes were analysed using the UCSF Chimera visualisation software.
